# Commentary: Adenosine A_2A_ Receptor Blockade Prevents Rotenone-Induced Motor Impairment in a Rat Model of Parkinsonism

**DOI:** 10.3389/fnbeh.2017.00093

**Published:** 2017-05-19

**Authors:** Marina F. de Souza, José M. M. Bispo, Pollyana C. Leal, Auderlan M. de Gois, José R. dos Santos

**Affiliations:** ^1^Department of Physiology, Federal University of SergipeSão Cristovão, Brazil; ^2^Behavioral and Evolutionary Neurobiology Laboratory, Department of Biosciences, Federal University of SergipeItabaiana, Brazil

**Keywords:** Parkinson disease, adenosine, neuroprotection, caffeine, dopamine

A recently published paper by Fathalla et al. (2016), demonstrated a rotenone induced possible protective effect of ZM241385 (a selective A2A receptor antagonist), but not of 8-cyclopentyl-1,3-dipropylxanthine (a selective A1 receptor antagonist), in a rat model of Parkinson's disease (PD). In the present paper, the discussion is short and presents few details. This commentary aimed to emphasize certain fundamental issues involving the rotenone model, the neuroprotective capacity of A2A receptor antagonists and compensatory mechanism of the non-dopaminergic approach for the treatment of PD.

Rotenone is the most potent member of the Rotenoids, a family of a natural flavonoids obtained from roots of tropical and subtropical plants belonging to the genus *Lonchocarpus* and *Derris* (Alam and Schmidt, 2002). Despite some limitations regarding variability and reproducibility seen in the animal model of PD induced by rotenone (Cannon et al., 2009), this model seems to replicate many hallmarks of illness including α-synuclein aggregation and Lewy body formation (Martinez and Greenamyre, 2012). Rotenone has lipophilic nature, and this feature induces nigrostriatal degeneration because rotenone inhibits complex I of the mitochondrial electron transport chain, decreasing ATP production, which can form reactive oxygen species such as superoxide, and reduced glutathione levels cause oxidative stress and cell death (Duty and Jenner, 2011; Johnson and Bobrovskaya, 2015). Fathalla et al. (2016) showed a progressive model of PD induced by six subcutaneous injections of rotenone. In this model, animals exhibited motor deficits as well as reduced level of dopamine in the midbrain.

To date, there is no efficient strategy to block or prevent the PD progression. It is expected that new drugs would stop the disease by a neuroprotective action. Thereby, A2A receptor antagonists represent a new way forward in the treatment of pathology (Roshan et al., 2016). All subtypes of adenosine receptors have been found in the central nervous system. The adenosine A2A receptors are abundant in striatum as well as in nucleus acumbens, where they are always co-localized with the dopaminergic D2 receptors (Perez-Lloret and Merello, 2014). In striatum, A2A receptors are localized mainly postsynaptically, but also presynaptically, on the neuron body and on glia cells (Cieślak et al., 2008). The adenosine was identified as a modulator of dopaminergic neurotransmission, based on studies with adenosine receptor antagonists in the rat models of hemiparkinsonism (Navarro et al., 2016). These receptors also regulate the release of other neurotransmitters such as noradrenaline, glutamate, acetylcholine, and gamma-aminobutyric acid (GABA; Cieślak et al., 2008). Studies suggest that the mechanism of interaction between the dopamine and adenosine receptors leads to changes in the affinity and coupling of G proteins, modulating the receptor efficacy. Thus, on stimulation of the adenosine receptor, the affinity between the dopaminergic agonists and DA receptors is reduced; hence, the adenosine agonists have similar effects to that of the dopaminergic antagonists. Alternatively, the effects of adenosine antagonists are similar to those produced by the dopaminergic agonists (Fuxe et al., 1998; Prediger et al., 2005).

In this study, Fathalla et al. demonstrated that A2A receptor antagonist attenuated the motor impairments (assessed by stride length and grid walking test) induced by rotenone, whereas A1 receptor antagonists did not show significant effect. Besides, the authors showed that A2A receptor antagonists might prevent the dopaminergic neuronal loss in striatum. We agree with the authors when they affirm that multiple mechanisms may be involved in this process. The neuroprotective capacity of A2A receptor antagonists could be linked to the action of microglial and astroglial cells in striatum as well as to the cytokines release (TNF-α, IL-1β) (Daré et al., 2007). It is important to emphasize that A2A antagonist attenuated the motor alterations after the last injection of rotenone even with a reduced level of dopamine in this group. Previous studies have shown that the decreased level of dopamine in striatum leads to enhanced dopamine receptor density (Takahashi et al., 2016). We assume that the attenuation of motor impairments by A2A antagonists can be related to compensatory mechanism in dopaminergic receptor density. Moreover, the A2A receptors modulate the indirect basal ganglia pathway due their co-localization with the dopaminergic D2 receptors (A2A–D2). Thus, the effect of adenosinergic antagonists on this dopaminergic pathway is significantly increased due the enhanced A2A–D2 receptor density (Figure 1).

**Figure 1:**
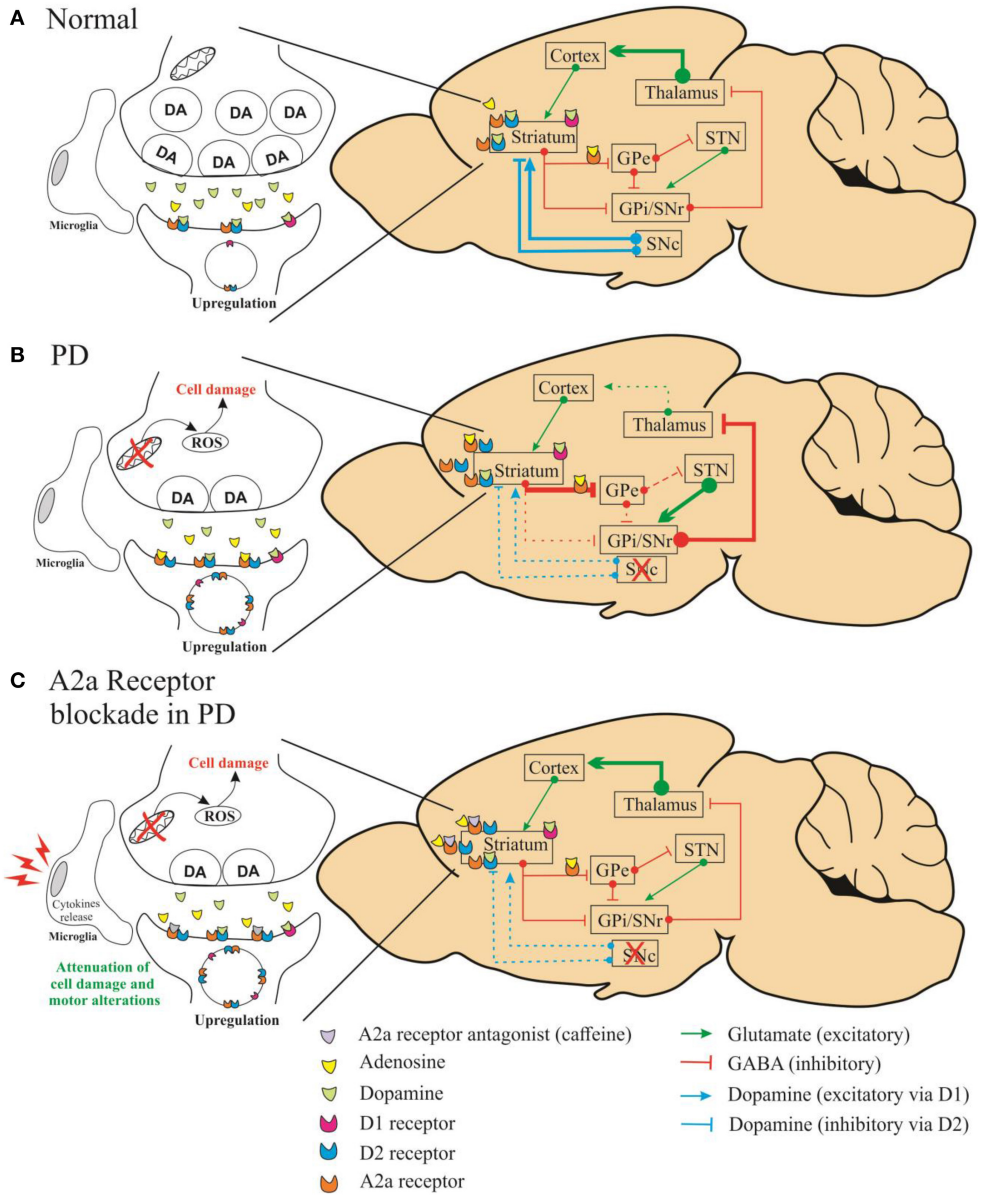
**Schematic representation of simplified diagram demonstrating the connections within the basal circuitry, and changes in the activity of basal ganglia nuclei associated with the mechanism of symptomatic anti-Parkinsonian activity of A2A receptor antagonists**. The striatum is connected to the thalamus through two pathways: indirect (striatum/globus pallidus pars externa-GPe/subthalamic-STN/nigral) and direct (globus pallidus pars interna complex-GPi/substantia nigra pars reticulate-SNr) pathways. In the normal state **(A)**, dopamine of the substantia nigra pars compacta (SNc) acts on inhibitory D2 receptors of the indirect pathway and on stimulatory D1 receptors of the direct pathway. In **(B)**, altered pathways are seen in Parkinson's disease caused by depletion of dopamine. In **(C)**, the A2A receptors modulate the indirect basal ganglia pathway due the co-localization with the dopaminergic D2 receptors (A2A–D2).

The neuroprotective effect of adenosinergic antagonists was demonstrated by other studies. Soliman et al. (2016) showed that the treatment with caffeine (adenosinergic antagonist) ameliorates the neuron loss in the substantia nigra pars compacta (SNpc), induced by rotenone. Besides, this study demonstrated that caffeine has a dose-dependent neuroprotective effect. Randomized controlled trial showed that caffeine may to represent a promising therapeutic tool in PD (Postuma et al., 2012); however, there are some limitations related to the atypical dose of caffeine used to improve the motor symptoms. High caffeine intake causes hyperactivity, which affects the basic and fundamental human process. Furthermore, researches are needed to elucidate the underlying mechanism related to the neuroprotective potential of adenosinergic antagonists for the treatment of PD.

## Author contributions

All authors listed, have made substantial, direct and intellectual contribution to the work, and approved it for publication.

### Conflict of interest statement

The authors declare that the research was conducted in the absence of any commercial or financial relationships that could be construed as a potential conflict of interest.
